# Hemodynamic Scaling of Task-Induced Signal Changes in Tumor Subjects

**DOI:** 10.3389/fnhum.2020.569463

**Published:** 2020-10-02

**Authors:** Tianming Qiu, N. U. Farrukh Hameed, Ching-Po Lin, Bharat B. Biswal, Jinsong Wu

**Affiliations:** ^1^Department of Neurosurgery, Huashan Hospital, Shanghai Medical College, Fudan University, Shanghai, China; ^2^Neurosurgical Institute of Fudan University, Shanghai, China; ^3^Shanghai Clinical Medical Center of Neurosurgery, Shanghai, China; ^4^Shanghai Key Laboratory of Brain Function and Restoration and Neural Regeneration, Shanghai, China; ^5^Institute of Neuroscience, National Yang-Ming University, Taipei, China; ^6^Institute of Science and Technology for Brain-Inspired Intelligence, Fudan University, Shanghai, China; ^7^Department of Biomedical Engineering, New Jersey Institute of Technology, Newark, NJ, United States

**Keywords:** fMRI, hemodynamics, brain tumor, breath-hold, motor, language

## Abstract

**Background:** FMRI signal amplitude can change during stimulus presentation due to underlying neural function and hemodynamic responses limiting the accuracy of fMRI in pre-surgical planning. To account for these changes in fMRI activation signal, we used breath-hold tasks to mimic hemodynamic changes in brain tumor subjects and scaled the activation response.

**Methods:** Motor and/or language fMRI was performed in 21 subjects with brain tumor. A breath-hold task was also performed in these subjects to obtain the hemodynamic response changes independent of neural changes. The task activation signals were calibrated on a voxel wise basis for all the subjects. Direct cortical stimulation was used to verify the scaled results of task-based fMRI.

**Results:** After scaling for the hemodynamic response function (HRF) on a voxel wise basis, the spatial extent of the scaled activation was more clustered together and appeared to minimize false positives. Similarly, accounting for the underlying canonical HRF, the percentage increase of active voxels after scaling had lower standard non-deviation suggesting that the activation response across voxels were more similar.

**Conclusion:** Although preliminary in nature, this study suggests that the variation in hemodynamic changes can be calibrated using breath-hold in brain tumor subjects and can also be used for other clinical cases where the underlying HRF has been altered.

## Introduction

Blood oxygenation level dependent (BOLD) based fMRI is a non-invasive method for mapping human brain function by indirectly measuring neural activity. Compared to direct cortical stimulation, the sensitivity and specificity of motor fMRI are 78.57–100% and 68–87%, respectively, while the sensitivity and specificity of language fMRI are 37.1–80% and 68–83.4%, respectively (Bizzi et al., 2008; Meier et al., 2013; Qiu et al., 2014; Kuchcinski et al., 2015). False positive and negative results in fMRI signal due to compromised cerebrovascular changes that accompany brain tumors makes reliable task-activation signal detection difficult. In addition, these results can result in inadequate resection of tumors and false negative results could lead to damage of functional areas. We hypothesized that these false positive or negative activation changes are due to compromised cerebrovascular changes that accompany brain tumors and this could reduce or enhance fMRI signal, making reliable task activation signal detection difficult.

To overcome the challenge of large variations in hemodynamic characteristics existing between subjects and even between brain regions in fMRI-BOLD response, a number of research groups have attempted to simulate regional hemodynamic differences by inducing physiological perturbations. Methods ranging from inhalation of gas mixtures to injection of acetazolamide have been used to stimulate hemodynamic activity without significantly altering neural activity (Kastrup et al., 1999; Li et al., 1999; Kannurpatti et al., 2002). Another such physiological perturbation was the induction of mild to moderate hypercapnic response via intermittent breath-hold or inspiration of a CO_2_/air mixture. These hypercapnic methods permit scaling of task-induced signal changes to account for regional vascular differences (Bandettini and Wong, 1997; Cohen, 1997). The understanding of implications of variation in hemodynamic changes on fMRI signal remains incomplete in patients with brain tumors. Calibrating for these changes could enhance the clinical applications of fMRI.

In this study, we generate cerebrovascular reactivity maps from breath-hold tasks in addition to task activation maps from motor and language tasks. We also generate a calibrated activation map that is then normalized on a voxel wise basis to account for differences due to cerebrovascular reactivity. Furthermore, we explain the importance and applicability of scaling to better understand the signal mechanism.

## Methods

### MRI Acquisition

Preoperative enhanced T1-weighted images (T1WI) and T2-weighted fluid-attenuated inversion recovery images were acquired using a 3.0-T interventional MRI scanner using standard parameters (Siemens Medical Solutions, Erlangen, Germany). Preoperative images were obtained 1–2 days prior to the date of surgery. Enhanced T1-weighted images (T1WI) were acquired to differentiate patients with high-grade or low-grade gliomas using the following imaging parameters: TR = 1,900 ms; TE = 2.93 ms; flip angle = 90°; number of slices = 176; slice thickness = 1 mm; and field of view (FOV) = 250 × 219 mm. The T2-weighted fluid-attenuated inversion recovery (T2-FLAIR) images were acquired with multi-shot Turbo Spin Echo (TSE) sequences with TR = 9,000 ms, TE = 99 ms, TI = 2,500 ms, flip angle = 150°, number of slices = 66, slice thickness = 2 mm, and FOV = 240 × 214 mm. Functional MRI (fMRI) data were acquired using a single-shot echo-planar imaging (EPI) sequence (TR = 3,000 ms; TE = 30 ms; flip angle = 90 degrees; slice number = 46; FOV = 240 × 240 mm; voxel size = 2.5 × 2.5 × 3 mm^3^). Each of the tasks and breath holding fMRI data acquisition lasted for about 3 min. Each run was preceded by 8 s of dummy scans for magnetization stabilization.

### Subjects

The study was conducted on patients with a primary lesion in the frontal, parietal, temporal, or insular lobe near (or involving) the motor or language cortical areas. The study was approved by the Institutional Review Board and all subjects provided consent to the study.

### Motor Task

Participants sequentially opened and closed both fists in rapid succession at a frequency of once cycle per second to the best of their ability. The finger-tapping paradigm consisted of three repetitions of bilateral finger tapping for 30 s and 30 s of rest. The scanning began with 30 s of rest scan.

### Language Task

Participants performed a picture-naming task while images were projected to the screen for 3 s. Subjects were instructed to quietly name the picture without speaking out. The language paradigm was identical to the finger-tapping paradigm and consisted of three repetitions of 30 s of picture naming and 30 s of rest.

### Breath-Hold Task

The breath-hold experiment consisted of a 30-s rest period (normal breathing) followed by three repetitions of 16 s of breath-hold and 44 s of normal breathing. Subjects performed an end-inspirational breath-hold, inhaling a similar volume of air to what they would in a normal breathing cycle (Kannurpatti et al., 2002; Biswal et al., 2007). During the breath holding task, the subjects were instructed to keep their eyes open. Subjects were trained on the breath-hold technique a few minutes prior to the actual scanning session.

### Data Analysis

All fMRI data sets were preprocessed using AFNI (Cox and Hyde, 1997). For each run, motion was estimated for each image with respect to a reference image along all the six planes (x, y, z, xy, yz, xz). The calculated mean for the displacement along the x-, y-, and z-axis is shown in Table 1. All data sets exhibiting head motion were corrected prior to further analysis or discarded if motion-induced artifact exceeded one voxel-shift. All data sets were corrected for linear trends. Standard deviation (SD) of the voxel time course was used as a measure of signal variability. Maps of the SD were obtained during breath-holding. Any subject with motion >2 mm were excluded from any further analysis.

**Table 1:** Motion parameters.

**No.**	**x-displacement**	**y-displacement**	**z-displacement**
1	0.0028	0.0102	0.005
2	0.0059	0.011	0.0039
3	0.0096	0.0121	0.0114
4	0.0161	0.0866	0.0024
5	0.0026	0.0369	0.004
6	0.0036	0.0392	0.0066
7	0.0066	0.0458	0.0071
8	0.0037	0.0448	0.0108
9	0.0057	0.0465	0.0187
10	0.0158	0.0376	0.0303
11	0.0184	0.059	0.0413
12	0.0018	0.0494	0.0392
13	0.0032	0.0542	0.0414
14	0.071	0.041	0.0342
15	0.0374	0.1123	0.0422
16	0.0449	0.1163	0.0566
17	0.0506	0.1351	0.0417
18	0.0873	0.1532	0.0321
19	0.1021	0.1101	0.0584
20	0.0737	0.1371	0.0537
21	0.0621	0.1464	0.0484

*The motion parameters from all the 21 subjects during the breath hold scan is tabulated. Motion was estimated using a 3D volume registration method. Motion parameters were estimated along all the six parameters. The motion parameters were found not to be significant in any of the subjects during the breath hold scan. The mean displacement along the x-, y-, and z-plane is displayed*.

### Functional MRI Analysis

An idealized time-course representing an “ON/OFF” stimulus was used as a reference waveform for cross-correlation analysis to identify stimulus-locked responses (Bandettini et al., 1993). Inherent to the cross-correlation technique is the assumption that neuronal activity and the fMRI signal vary synchronously with the stimulus paradigm. Cross-correlation analyses identified voxels that had a shape similar to that of the reference waveform. If we represent the reference waveform (with a mean value of μ_*r*_) by *r*_*i*_ and the voxel time-course (with a mean value of μ_*t*_) by *t*_*i*_, where *i* = 1,2,3 … *N* are the number of data points, the correlation-coefficient between the reference waveform *r*_*i*_ and the voxel time course *t*_*i*_ may be written as cycle per second to the best of (Bandettini et al., 1993):

(1) $$\begin{array}{r} cc=\frac{\overset{N}{\underset{i-1}{\sum }}\left(t_{i}-\mu_{t}\right)\left(r_{i}-\mu_{r}\right)}{\sqrt{{\overset{N}{\underset{i=1}{\sum }}\left(t_{i}-\mu_{t}\right)}^{2}{\overset{N}{\underset{i=1}{\sum }}\left(r_{i}-\mu_{r}\right)}^{2}}} \end{array}$$

For each scan, a histogram of the cross-correlation coefficients throughout the entire brain during rest was used to select a threshold coefficient for a valid response. Typically, correlation values > 4 times the SD of the resting state cross-correlation coefficient distribution were considered active voxels and their locations noted. Using this criterion, a threshold correlation coefficient 0.4 guaranteed statistical significance (*p* < 0.001) after including a correction for multiple comparisons. The significance of clusters was determined with AFNI's 3dClustSim using mixed autocorrelation function (ACF) modeling to account for the spatial smoothness of noise at a voxel wise significance threshold of *p* = 0.005 and cluster wise significance of α < 0.05 (with 5,000 Monte Carlo simulations).

### Gamma-Variate Analysis

The breath hold data was fitted using the gamma-variate model on a voxel wise basis. The gamma-variate fit was optimized for each voxel time course. As a consequence, different gamma-variate fit parameters were obtained for each voxel in the brain. The onset time of the breath hold signal *t*_0_ was determined from a gamma-variate fit to the image data on a voxel-wise basis (**Figure 3**). The gamma-variate function is defined as:

(2) $$\begin{array}{r} S\left(t\right)=Q{\left(t-t_{0}\right)}^{\text{r}}e^{-\left(\text{t}-{\text{t}}_{0}\right)/\text{b}}\; \end{array}$$

where *S(t)* is the MR signal intensity, *Q, r, b* are fit constants, *t* is the time after injection, and *t*_0_ is the appearance time of the tracer (Zierler, 1965a,b). The *t*_0_ value was determined for all voxels in the cortex, resulting in the generation of a *t*_0_ map. In a similar fashion, Q, r, b, S_max_, T_max_, and S_Area_ maps were also generated. The breath-hold paradigm in this study was assumed to follow the tracer kinetics model. In a study by Cohen (Cohen, 1997), very brief visual stimulus (for 1 s) was presented several times and fMRI response was averaged and fitted with a Gamma-variate fit to obtain the impulse response function. Similarly, in this study the breath hold response was fitted to the gamma variate model, and several parameters including t_0_, r, b were considered as the onset time of the fMRI response following the stimulus, while t represented the sampling rate. Non-linear regression was used to estimate the gamma-variate fit parameters using AFNI. Briefly it estimated the gamma-variate fit parameter using a predefined number (set as 1,000 for this study) with signal and noise constraints. From the 1,000 values of the parameters obtained, values from the five best fit parameters were selected. This was repeated 1,000 times to avoid any local minima that the algorithm may face and also to minimize for any bias in the estimation. With the limited low SNR of the fMRI time series, this provided another reason to use a large number of estimates using non-linear regression.

To calculate the reliability of the estimation of the gamma-variate fit parameters, the breath-hold data sets containing the three ON/OFF cycles were broken into three scans with one breath-hold epoch in each of the data set. Identical gamma-variate analysis was performed for each data sets and a correlation analysis was performed between each of the gamma-variate fit parameter from the first data set with the corresponding parameter in the second data sets. The breath holding data for the three cycles were assumed to be similar across all the three runs. This was simply assuming linearity about the fMRI signal. Identical gamma-variate analysis was performed for each of three data sets and the gamma-variate fit parameters were compared across all the voxels. A correlation analysis was performed between each of the gamma-variate fit parameter from the first data set with the corresponding parameter in the second data sets. A bootstrap resampling method was used to test the reliability and confidence interval of the gamma-variate fit parameters. The parameters obtained from each of the three ‘pseudo” runs were randomly selected to compute the correlation coefficient between the parameters. This process was repeated one thousand times. Significant difference between the scaled and unscaled conditions was tested using the Bartlett's test of homogeneity of variance.

### Task Activations

#### Motor/Language Task

In the generalized linear model of motor/language task, the periods of finger tapping/picture naming duration of 30 s were modeled as a box-car function, and convolved with a canonical hemodynamic function. The generalized linear models were estimated in a voxel-wise manner and the beta map representing motor/language task activations was obtained for all tasks.

Task-activation data sets for finger tapping/picture naming were processed in a similar fashion. An ideal box-car waveform corresponding to the stimulus presentation was cross-correlated with every pixel time-course on a pixel-by-pixel basis for all the subjects. Histograms of the cross-correlation coefficient values during rest were used to select a minimum criterion for valid response. Typically, correlation values were considered valid if they were > 4 times the SD of the resting state distribution. To determine how the BOLD signal response scaled in every subject during the finger tapping tasks, the distributions of the BOLD signal change were obtained before and after scaling.

#### Breath-Hold Task

In the generalized linear model of breath hold, breath-hold periods were modeled as a box-car function with an onset delay for 8 s to account for the BOLD response delay of breath-hold (Biswal et al., 2007). Then, the delayed box-car function was convolved with hemodynamic function. After estimating the generalized linear model, beta map representing breath hold activations was obtained for future analysis.

Scaling was accomplished by dividing the BOLD signal response (beta weights) during the motor task and with the BH induced BOLD response amplitude (gamma-variate estimates) obtained for every voxel in the brain. Beta estimates of the BOLD signal amplitude change during finger tapping and language task were obtained from the GLM analysis (described above) during the motor task.

## Results

A total of 21 patients were included: 10 men and 11 women aged from 16 to 70 years (Table 2).

**Table 2:** General clinical data.

**No.**	**Age**	**Gender**	**Tumor type**	**Tumor location**
1	48	F	A	Left frontal
2	48	M	GBM	Left parietal
3	49	F	A	Left frontal
4	68	F	GBM	Left frontal
5	47	F	GBM	Left frontal and parietal
6	44	M	O	Right frontal
7	40	M	O	Left frontal
8	36	M	A	Left temporal and frontal
9	43	M	A	Left frontal
10	16	M	GBM	Left frontal
11	20	F	GBM	Right frontal, temporal
12	43	M	GBM	Right temporal and insular
13	62	F	A	Right frontal
14	70	F	GBM	Right temporal and insular
15	33	M	AA	Left frontal
16	44	F	GBM	Right frontal
17	31	F	GBM	Left frontal
18	27	M	A	Left frontal and insular
19	30	F	A	Right frontal
20	44	M	A	Left frontal
21	43	F	O	Left frontal

*M, male; F, female; A, astrocytoma; O, oligodendroglioma; AA, anaplastic astrocytoma; GBM, glioblastoma multiforme*.

* *2016 WHO classification of tumors of the central nervous system*.

Significant amount of signal changes were observed in all subjects during breath-hold. After breath-hold of about 15 s, the signal intensity was observed to increase. An idealized input waveform corresponding to the ON/OFF paradigm was shifted by 15 s (to account for the delay in response) and correlated with every voxel time series in the brain. All voxels with a correlation coefficient >0.4 were considered active and classified as gray matter (correlation values were considered valid if they were >4 times the standard deviation of the resting state distribution). Also, a significant increase in gray matter was observed compared to white matter. The percent change in gray matter was significantly higher than in white matter region. The mean percent signal change corresponding to the breath-hold stimulus was observed in all the subjects during breath holding. Table 3 shows the delay onset time (to), mean gray and white matter changes in each subject.

**Table 3:** Gamma-variate parameters.

**No.**	**To (sec)**	**Gray matter**	**White matter**
1	24.75 ± 7.28	0.18 ± 0.07	0.06 ± 0.22
2	22.47 ± 5.44	0.19 ± 0.08	0.04 ± 0.02
3	24.30 ± 5.72	0.21 ± 0.09	0.02 ± 0.02
4	23.32 ± 5.32	0.37 ± 0.12	0.14 ± 0.07
5	22.81 ± 8.14	0.23 ± 0.06	0.07 ± 0. 03
6	22.24 ± 6.47	0.24 ± 0.09	0.05 ± 0.04
7	24.84 ± 7.84	0.42 ± 0.15	0.08 ± 0.07
8	23.54 ± 6.49	0.19 ± 0.07	0.03 ± 0.05
9	24.36 ± 7.16	0.26 ± 0.08	0.04 ± 0.03
10	22.98 ± 3.17	0.17 ± 0.14	0.05 ± 0.04
11	23.27 ± 4.65	0.31 ± 0.21	0.06 ± 0.07
12	24.13 ± 4.27	0.25 ± 0.08	0.08 ± 0.04
13	22.52 ± 4.76	0.27 ± 0.11	0.11 ± 0.08
14	21.65 ± 3.83	0.34 ± 0.17	0.09 ± 0.07
15	23.28 ± 4.28	0.16 ± 0.12	0.03 ± 0.03
16	21.52 ± 4.18	0.25 ± 0.08	0.04 ± 0.02
17	23.14 ± 3.14	0.16 ± 0.09	0.02 ± 0.05
18	22.67 ± 3.71	0.27 ± 0.10	0.07 ± 0.02
19	24.18 ± 3.82	0.34 ± 0.12	0.07 ± 0.03
20	24.21 ± 3.67	0.42 ± 0.15	0.11 ± 0.02
21	24.75 ± 7.28	0.38 ± 0.09	0.09 ± 0.07

*The onset time (To) along with the mean signal change in gray- and white-matter motion parameters from all the 21 subjects during the breath hold scan is shown here*.

**Figure 1:**
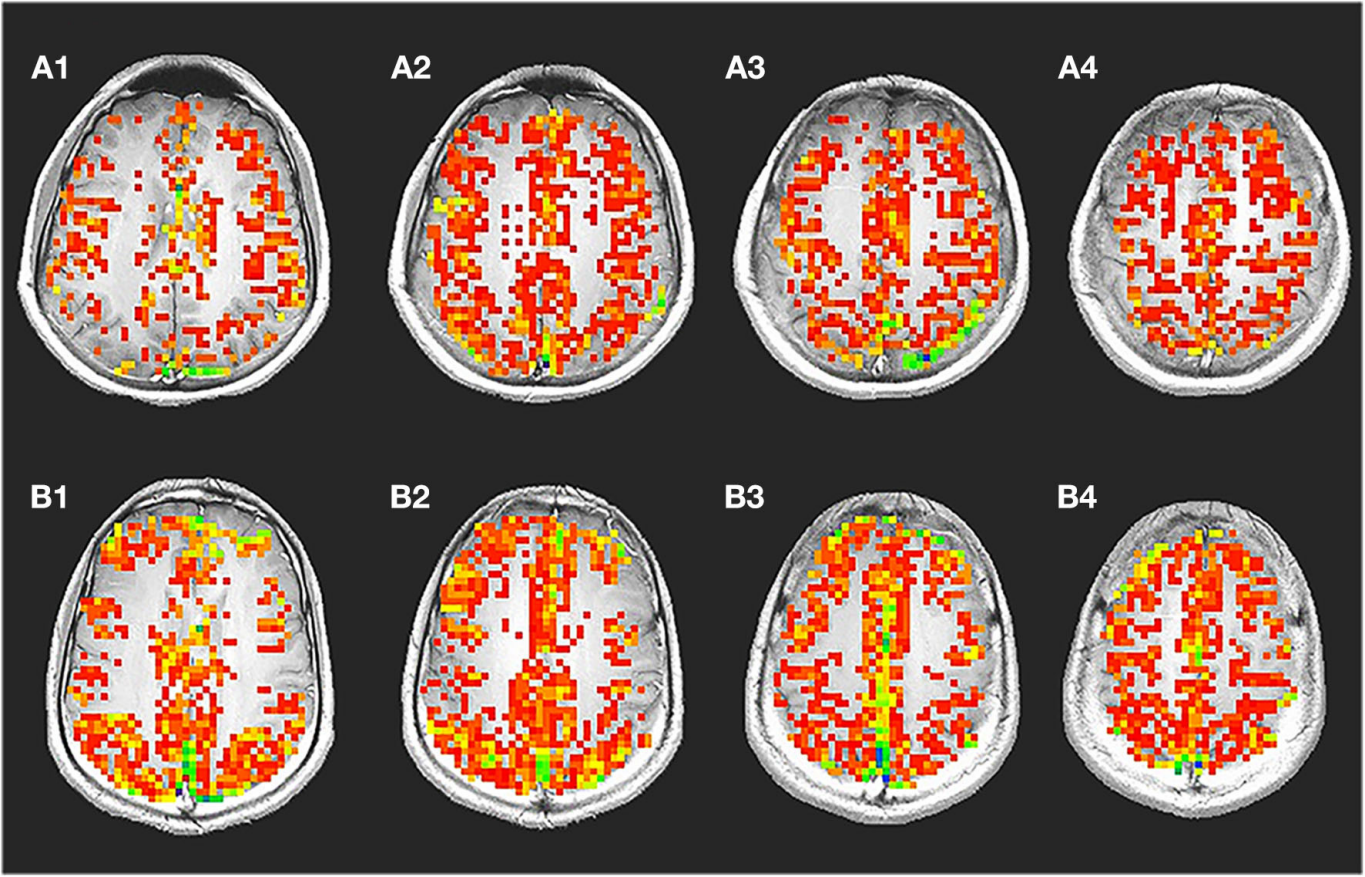
Breath-hold activation maps, ideal box paradigm with time series. A representative spatial map **(A)** showing voxels with significant changes during breath hold time series **(B)** is shown. During breath hold a significant change was observed in the gray matter region. Significantly less number of voxel from the white matter regions passed the threshold. The time series shows signal intensity changes in the gray matter during breath hold. It was typically seen that, the voxel time series from the gray matter changed by about 2.386%. In the white matter though, a significantly lower amount of 0.945% change was also observed.

Figure 1A shows a representative voxel time course from the gray matter regions: signal changes corresponding to changes in the stimulus was observed in voxels from gray matter. Figure 1B shows corresponding maps of images that correlated significantly with the idealized reference box-car function representing the breath-hold timing.

**Figure 2:**
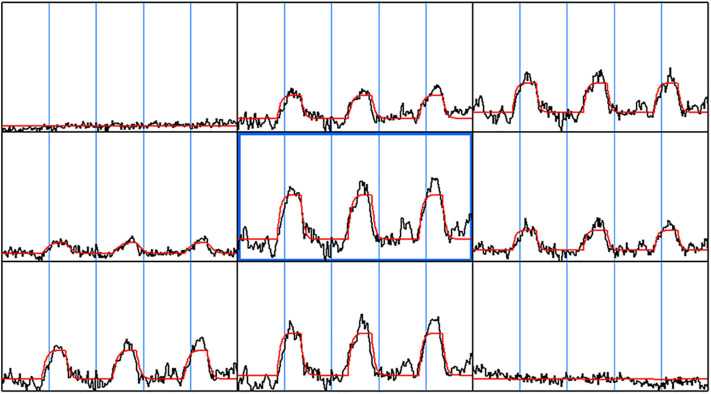
Gamma-variate fit. The gamma-variate fit parameters including t0, a, b, r were computed on a voxel by voxel basis for the entire brain. A 3 × 3 window of neighboring voxel time series along with the gamma-variate fit is shown. As can be seen, the gamma-variate fit was able model the actual response from each of the epochs. Also, in the voxel time series where there was no signal change during breath hold task, the gamma-variate fit gave a good fit—a straight line.

The gamma-variate analysis was next used to fit every voxel time series in the brain and each fit parameter was then estimated. Figure 2 shows representative voxel time series along with the gamma-variate fit parameters: Maps of the onset time (t_0_), time to peak (T_max_), maximum signal amplitude (S_max_)and total area covered by the signal during the stimulus (S_Area_), r, and b. A number of gamma-variate fit parameters including S_max_, S_Area_, t_0_, T_max_, r, and b showed good demarcation between the gray and white matter region. In a few cases regions from large vessels could be demarcated.

Significant differences in the mean of parameters including t_0_, amp, r, b, between gray matter and white matter regions for each of the subjects were observed. Significant difference was observed between the gray matter, white matter, and large vessels values for various parameters. Signal area (S_Area_) and t_0_ showed the greatest difference between the two regions. The ratio between the gray matter and white matter for signal areas varied between 3.11 and 2.09 with a mean value of 2.56. Similarly, the onset time for all the subjects varied between 16.1 and 11.4 s with a mean value of 13.7 s.

The correlation coefficient (estimate of reliability) for a representative subject (case 17) was found to be 0.8804±0.0110.

We were able to detect motor and/or language activation in all subjecs. The spatial extent of the scaled activation was clustered together. In particular, many of the voxels not clustered around the sensorimotor cortex and from regions close to large vessels after scaling did not pass the threshold. Similarly, the percentage increase of active voxels after scaling had lower SD suggesting that after taking into account the underlying HRF, the activation response across voxels were more similar.

### Case 1

**Figure 3:**
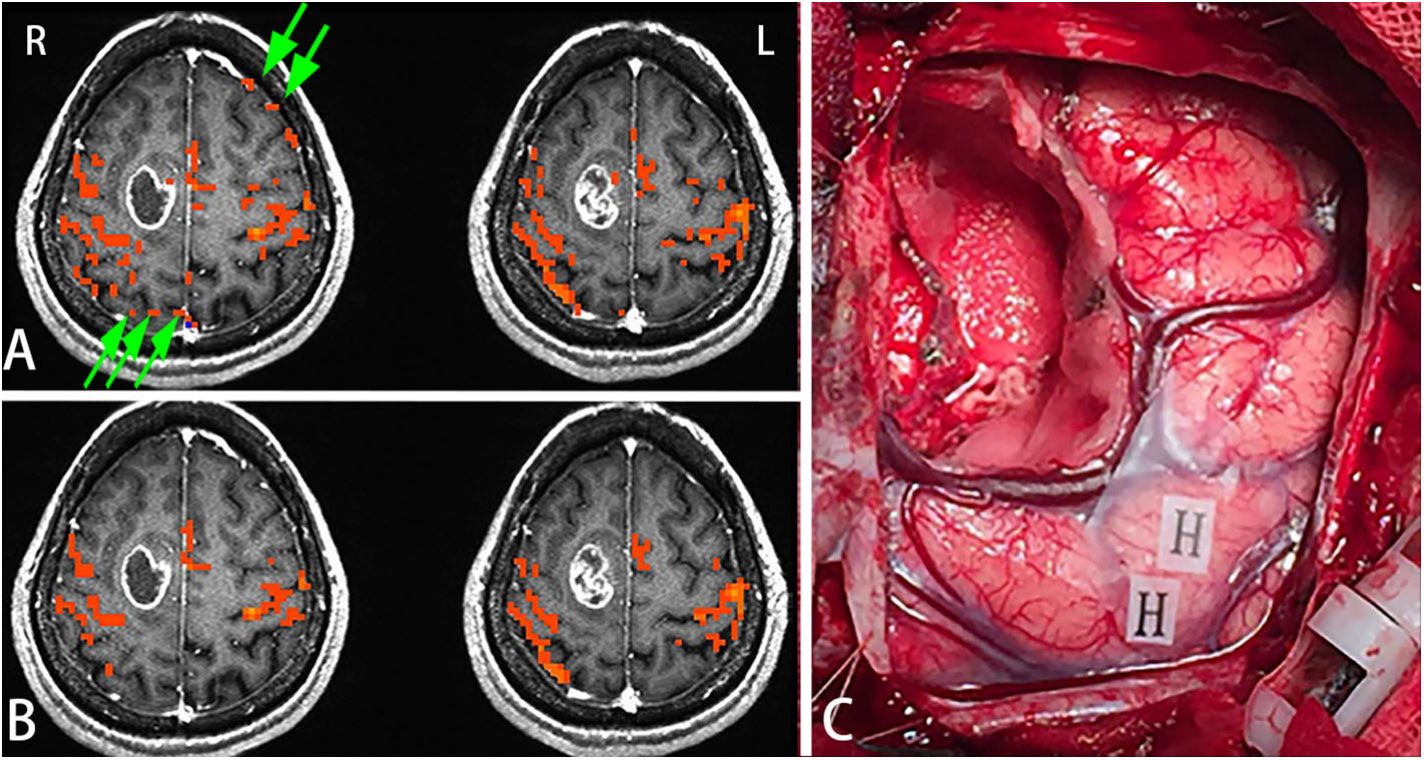
In this figure, the active voxels in addition to being in the sensorimotor cortex were dispersed in other brain regions **(A)**. After scaling the response using the BH task, the active voxels were clustered around the sensorimotor cortex. Very few voxels outside the sensorimotor cortex were observed suggesting that scaling can be used not only for removing false positives but also for increasing true detection **(B)**. Brain mapping results confirmed the motor area (H: hand motor area) which was shown on the scaled functional activation map **(C)**. Green arrows in A showed the false positive activities before scaling.

Female, 44 years, (case 16 in Table 1) seizures of left lower limbs for 1 month with normal muscle strength. MRI scans revealed tumor in the right frontal lobe (Figure 3A). In this subject, sensorimotor activation was obtained. After scaling the response using the breath-hold task, the active voxels were clustered around the sensorimotor cortex (Figure 3B). Brain mapping results confirmed motor area as shown on the scaled functional activation map (Figure 3C).

### Case 2

**Figure 4:**
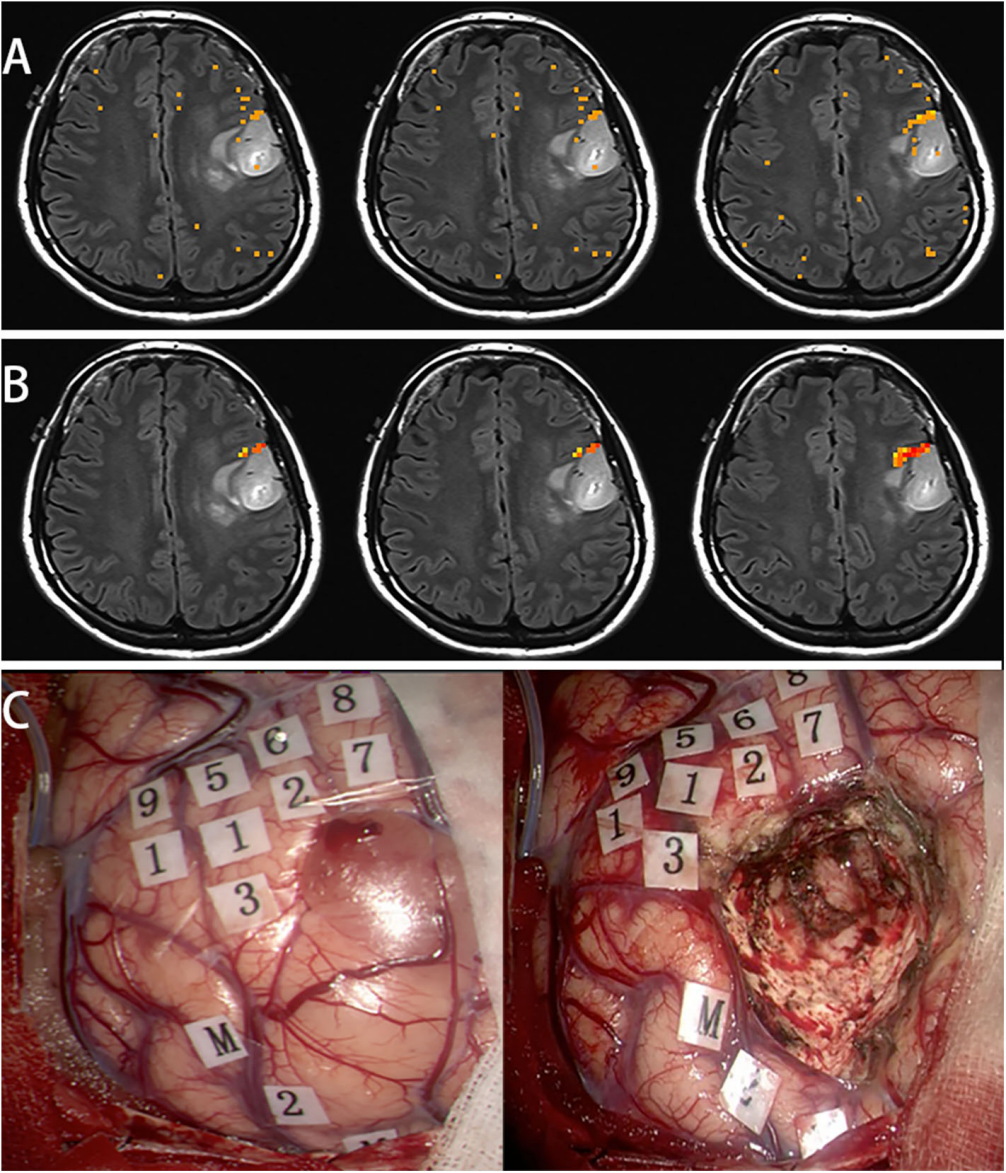
In this subject, a picture naming task was used to activate the Broca's area and other language associated regions **(A)**. After scaling using the breath hold response, the activation was clustered around Broca's area **(B)**. Awake surgery was performed for this patient. After brain mapping, several positive language sites were found, that was shown on the scaled functional activation map (**C**, 1-9: language area, M: mouth motor area).

Female, 31 years (case 17 in Table 1) epilepsy once with normal language function. MRI scans revealed tumor in the left frontal lobe (Figure 4A). In this subject, a picture-naming task was used to activate the Broca area and other language associated regions. After scaling using the breath-hold response, activation was clustered around Broca's area (Figure 4B). Brain mapping, confirmed multiple language sites as shown on the scaled functional activation map (Figure 4C).

**Figure 5:**
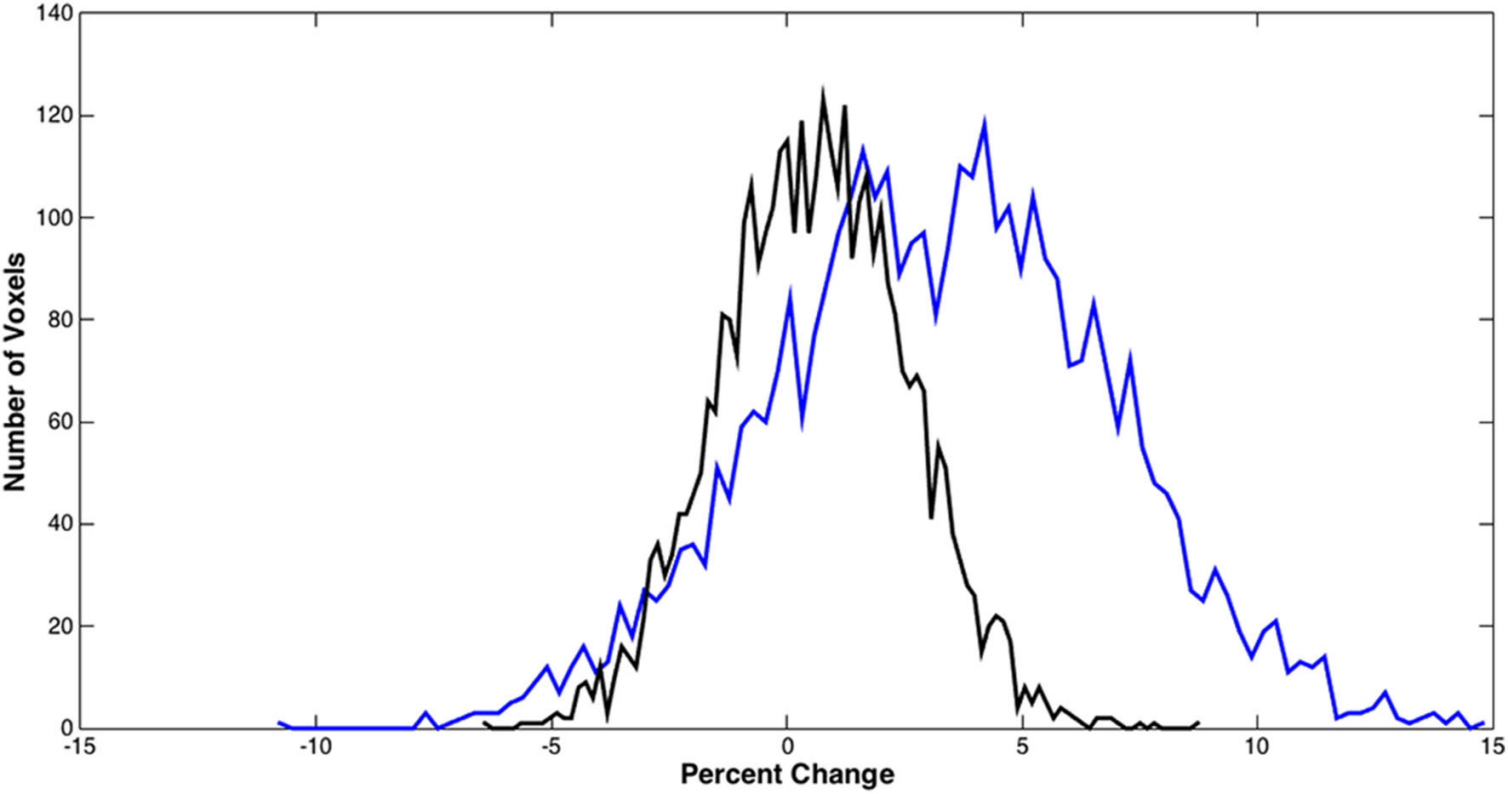
BOLD signal distributions during bilateral finger tapping from a representative subject. Histogram of the percent signal change before and after BH scaling in the sensorimotor cortex is shown. A significant reduction in the number of voxels with large percent change was observed.

Figure 5 shows the distributions of the BOLD signal change from the sensorimotor cortex in a representative tumor subject. The median of the distributions after scaling with breath-hold was reduced. Taken together with results from Figure 3, the finger tapping signal change showed greater variability prior to scaling. Similarly, in Figure 4 the language associated regions activation signal also showed greater variability compared to the scaled response.

## Discussion

First, we used breath-hold for scaling task activation in brain tumor subjects. From the data, segmentation between gray and white matter was used to scale activation response. The breath-hold data also identified large vessels present in the time series datasets.

In this study, the inter-voxel whole brain regression method used showed activations on BOLD maps in both bilateral finger tapping and language tasks. Results were consistent with previous literature on BOLD calibration (Kannurpatti et al., 2002; Biswal et al., 2007; Thomason et al., 2007), however, previous studies were exploratory, and task-related regions were not clearly delineated. By calibrating intra-subject BOLD variability across the whole brain, we investigated the possibility of detecting brain regions vulnerable to neuro-vascular coupling and regions in need of enhancement due to poor neuro-vascular response.

Regions with suppressed activations generally coincided with regions that had large neural-vascular response; implying the proposed adjustment method could be a powerful tool to calibrate BOLD activation maps. An additional, but not exclusive factor, contributing to the adjustment were the large vessels, as the distribution of the adjustment suppression agreed well with the distribution of large vessels.

The physiological parameters used to obtain segmentation suggests that these can be used to segment fMRI data into gray and white matter regions. The t_0_, and S_Area_ parameters had the greatest demarcation between gray and white matter regions. Parameters that can optimize tissue segmentation further are being investigated. In the clinic, the above parameters could be used to differentiate between healthy and diseased tissue types.

Presently, the accuracy of BOLD fMRI is insufficient for pre-surgical planning (Bizzi et al., 2008; Qiu et al., 2014; Kuchcinski et al., 2015). However, a study found decrease in BOLD signal enhancement in tumors in patients and concluded that breath-hold can disclose differences in cerebrovascular response between normal and glioma tissue (Pillai and Zaca, 2011). These results may be attributed to the tumor-induced neurovascular uncoupling. We used breath-hold data to calibrate motor and language fMRI before brain tumor surgeries. The results of scaled motor fMRI and language fMRI were verified by map- the current golden standard.

The assumption with breath-holding is that it reflects the underlying vasculature without any significant changes in contribution due to neuronal activity. Thus, variations in hemodynamic properties between regions caused by task response can be scaled. Scaling decreases variability and reflects signal changes due to neural activity after accounting for variability due to vascular differences. In this study, scaling refers to the division of task-induced BOLD response by BOLD response during brief breath-hold hypercapnia in each voxel.

## Limitations

The method proposed in this study, while potentially useful, has some limitations. First, this study was performed on clinical subjects and compliance could be an issue. Breath-hold could be challenging and in such subjects, this calibration method cannot be used. Other hypercapnic stimulation methods like inhalation of 2% CO_2_ could be performed instead. Second, due to the number of subjects used, we could not perform statistical analysis to calculate the sensitivity and specificity of scaled fMRI results. However, we found very good results at the individual-patient level.

## Conclusion

The current study, for the first time, investigated functional brain mapping after calibrating task activation response using the magnitude of the breath-hold response. We demonstrate the applicability of this method in brain tumor subjects where HRF may be compromised in tumor and neighboring regions. Using the breath-hold based hypercapnic calibration, brain regions could be reliably identified to minimize false positives.

## Data Availability Statement

Requests for data sharing will be considered by the authors upon reasonable request.

## Ethics Statement

The studies involving human participants were reviewed and approved by Huashan Hospital Insitutional Review Board. The patients/participants provided their written informed consent to participate in this study.

## Author Contributions

TQ, NH, C-PL, BB, and JW conceived and designed the study, drafted and revised the manuscript, and performed data extraction and analysis.

## Conflict of Interest

The authors declare that the research was conducted in the absence of any commercial or financial relationships that could be construed as a potential conflict of interest.
